# Transport choice when travelling to a sports facility: the role of perceived route features - Results from a cross-sectional study in the Netherlands

**DOI:** 10.1186/s13102-015-0009-6

**Published:** 2015-06-30

**Authors:** Ellen L de Hollander, Eline Scheepers, Harm J van Wijnen, Pieter JV van Wesemael, Albertine J Schuit, Wanda Wendel-Vos, Elise EMM van Kempen

**Affiliations:** 1 National Institute for Public Health and the Environment, Centre for Nutrition, Prevention and Health Services, PO Box 1, 3720 BA Bilthoven, Netherlands; 2 Department of Health Sciences and EMGO institute for Health and Care Research, VU University Amsterdam, De Boelelaan 1085, 1081 HV Amsterdam, Netherlands; 3 National Institute for Public Health and the Environment, Centre for Sustainability, Environment and Health, PO Box 1, 3720 BA Bilthoven, Netherlands; 4 Department of the Built Environment, Technical University Eindhoven, PO Box 513, 5600 MB Eindhoven, Netherlands

**Keywords:** Car use, Walking, Cycling, Active transportation, Physical activity, Sports, Transport choice, Route features

## Abstract

**Background:**

Physical activity and sedentary behaviour are independently associated with health outcomes, where physical activity (PA) is associated with health benefits and sedentary behaviour is associated with health risks. One possible strategy to counteract sedentary behaviour is to stimulate active transport use. As monitoring studies in the Netherlands have shown that among sedentary people the proportion of adults who engage in sports (hereafter: sports practitioners) is 62.3%, sports practitioners seem a feasible target group for this strategy. Previous studies have generally reported associations between neighbourhood characteristics and active transport use. However, the neighbourhood covers only part of the route to a certain destination. Therefore, we examined the association between perceived route features and transport choice when travelling up to 7.5 kilometres to a sports facility among sports practitioners.

**Methods:**

For 1118 Dutch sports practitioners – who indicated that they practice a sport and travel to a sports facility – age 18 and older, data on transport choice and perceived features of the route to a sports facility were gathered. Participants were classified into one of three transport groups based on their transport choice: car users, cyclists and walkers. Participants were asked whether perceived route features influenced their transport choice. Logistic regression was used to model the odds of cycling versus car use and walking versus car use in the association with perceived route features, adjusted for potential confounders.

**Results:**

Perceived traffic safety was associated with lower odds of cycling (OR: 0.36, 95% CI: 0.15-0.86). Perceived route duration was associated with lower odds of both cycling (OR: 0.54, 95%CI: 0.39-0.75) and walking (OR: 0.60, 95%CI: 0.36-1.00). Perceived distance to a sports facility and having to make a detour when using other transport modes than the chosen transport mode were associated with higher odds of both cycling and walking (OR_range_: 1.82-5.21). What and who people encountered during their trip (i.e. visual aspects) was associated with higher odds of both cycling and walking (OR_range_: 2.40-3.69).

**Conclusions:**

Perceived traffic safety, duration, distance, detour, and visual aspects, when travelling to a sports facility were associated with transport choice. Therefore, the perception of route features should be considered when stimulating active transport use among sports practitioners.

**Electronic supplementary material:**

The online version of this article (doi:10.1186/s13102-015-0009-6) contains supplementary material, which is available to authorized users.

## Background

Physical activity (PA) is associated with health benefits, such as the prevention of chronic diseases and an improved quality of life. Moreover, increasing the amount of PA can result in additional health benefits [1]. Sedentary behaviour is associated with health risks, such as an increased risk of type 2 diabetes, all-cause and CVD mortality [2]. These two behaviours, i.e. physical activity (PA) and sedentary behaviour, are independently associated with health outcomes [3–5]. Therefore, it is important to stimulate PA in both physically active persons, who can increase their PA levels, as well as in sedentary persons.

In the Netherlands, monitoring studies have shown that among sedentary people the proportion of adults who engage in sports (hereafter referred to as: sports practitioners) is 62.3% [6]. This indicates that a substantial proportion of sedentary people, who gain the most by increasing their PA levels, are sports practitioners. Therefore, sports practitioners may constitute a specific target population to increase their physical activity levels and decrease their sedentary behaviour despite the fact that they are actively involved in sports.

One way to increase PA levels is to stimulate active transport use. Stimulating active transport use has become a popular policy strategy and can be carried out by replacing short-distance car trips with cycling or walking [7]. A distance up to 7.5 kilometres may be considered feasible; it represents a maximum of 30 minutes of cycling at an average speed [8]. However, one set of barriers that may hamper active transport use lies in the physical environment. Previous studies have reported an association between neighbourhood characteristics, such as accessibility of facilities, availability of cycling and walking paths, safety (traffic and crime), and the aesthetic quality of the built environment and active transport use for different trip purposes [9–11]. However, the neighbourhood covers only part of the route to a certain destination (also referred to as ‘trip purpose’ in travel surveys). Moreover, factors influencing active transport use differ by trip purpose [12]. Therefore, insight into route features and the association with active transport use for specific trip purposes is needed.

In this study, we focus specifically on the associations between perceived route features when travelling to a sports facility and transport choice among sports practitioners. Findings from our study may be important for public health, as it can give guidance for policy programmes such as the Dutch national policy programme ‘Sports and Physical Activity in the Neighbourhood’ that stimulates physical activity by making the healthy choice, the easy choice (e.g. providing sports fields in the neighbourhood and improving the infrastructure to enhance physical activity) [13, 14].

## Methods

### Study design

This study was part of the Dutch ‘impActs of actiVE traNsport in Urban Environments’ (AVENUE) project. The aim of the AVENUE project is to provide in-depth information on characteristics of short car and active (cycling and walking) transport trips and the feasibility of replacing short car trips with short trips by active transport by using a multidisciplinary approach, including a combination of qualitative (focus groups, policy analysis) and quantitative methods (systematic literature review, questionnaire and (secondary) data analysis). In this study, we used the data obtained from the questionnaire that was designed by the AVENUE project group. The questionnaire was distributed by IPSOS-Nederland [15] among a random sample from their internet panel (Scheepers CE, Wendel-Vos GCW, van Kempen EEMM, de Hollander EL, van Wijnen HJ, Maas J, den Hertog FRJ, Staatsen BAM, Stipdonk HL, Int Panis LLR, van Wesemael PJV, Schuit AJ: Perceived Accessibility is An Important Factor in Transport Choice — Results from the AVENUE Project, Submitted). IPSOS-Nederland applies the Personal Data Protection Act [15] and gathered the informed consent from the participants in this study. As an Institutional Review Board (IRB) approval is only needed when the daily life of participants is influenced or participants are required to perform specific actions, an IRB approval was not warranted and therefore not obtained. The data were anonymised prior to the moment that the AVENUE project group received the dataset from IPSOS-Nederland. The authors did not have access to any identifying information.

For 3,663 adults age 18 and older, data were collected using an online questionnaire administered during one calendar year that started in July 2012. Data included information about individual characteristics, environmental characteristics, trip purposes, transport mode, factors influencing transport choice separately for car, bicycle and walking, health, and lifestyle.

**Fig. 1:**
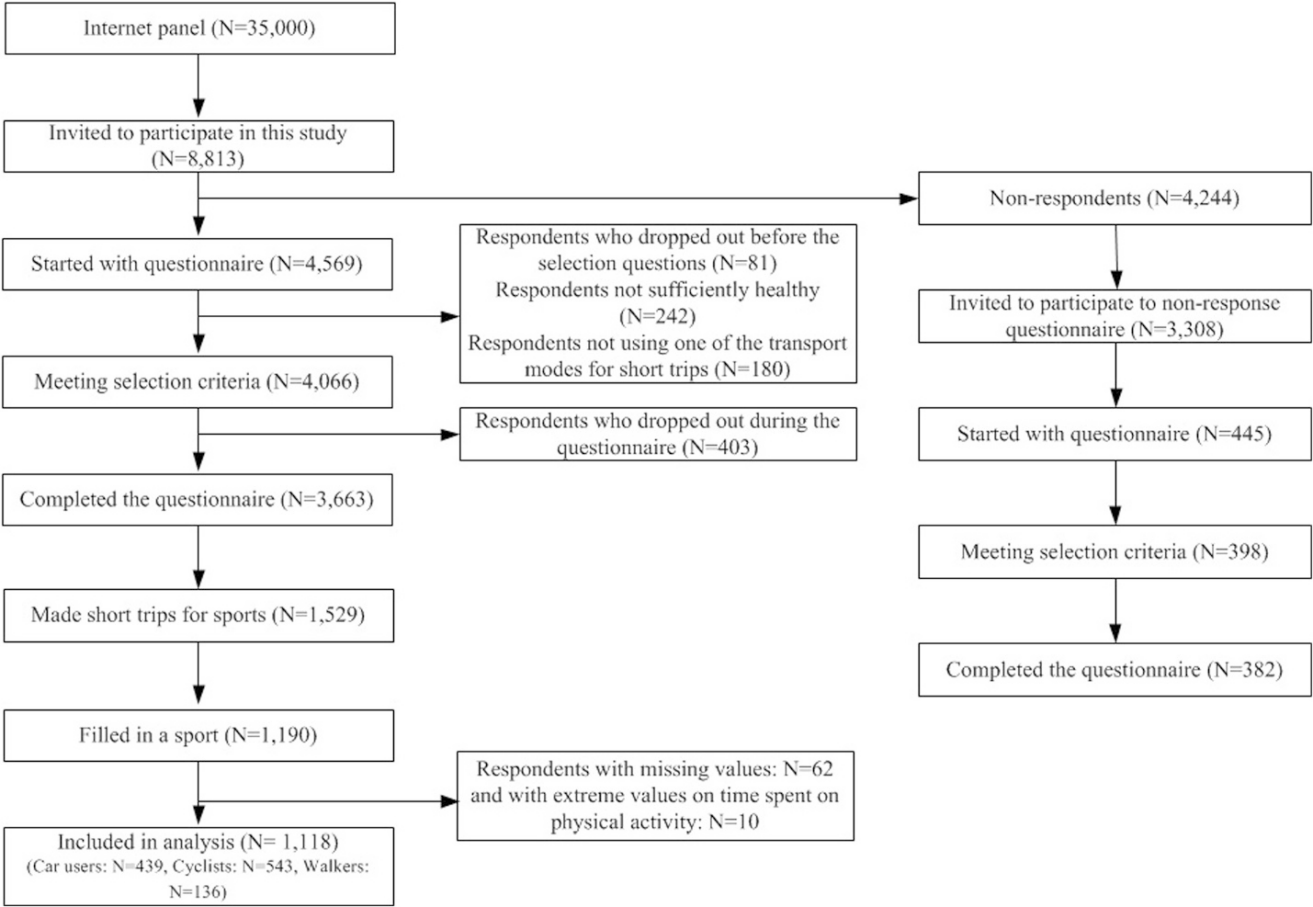
Flowchart of the study population

In this study, we focussed on route features when travelling to a sports facility. Thus, participants who answered that they travelled a distance up to 7.5 kilometres to a sports facility directly from home and who filled in a sport they practiced at least on a weekly basis were selected and defined as ‘sports practitioners’ (N = 1190; Fig. 1).

### Transport mode

Participants were classified into one of three transport groups based on their transport choice: car users (passive transport), cyclists and walkers (active transport). Their choice was inferred from their self-reported frequency of using the car, cycling, or walking when travelling to a sports facility. When participants used two or more transport modes equally frequent, they were categorised as a car user if one of the transport modes was a car, and as a cyclist in all other cases.

### Perceived route features

In the questionnaire, 13 items were included inquiring about the influence of route features on transport choice (see Table 1). These items included subjects with regard to safety, bother by noise, odour and vibrations, route convenience, and visual aspects. Answers were rated on a four-point category scale (‘(almost) never’, ‘sometimes’, ‘often’, ‘always’).

**Table 1:** Characteristics of the population by transport choice when travelling to a sports facility

	Car use (n = 439)	Bicycling (n = 543)	Walking (n = 136)	P_car vs cycling_	P_car vs walking_
Individual characteristics					
*Men, %*	53.3	49.0	55.2	0.18	0.71
*Age (yrs), mean (SD)*	48.4 (13.4)	45.7 (14.7)	46.6 (14.1)	<0.01	0.17
*Educational level, %*				0.22	0.98
High	35.5	40.7	34.6		
Medium	43.3	41.1	44.1		
Low	21.2	18.2	21.3		
*Household composition, %*				<0.01	0.03
living alone	17.3	21.6	26.5		
with a partner	39.0	37.0	36.0		
with children under 18	31.9	20.8	.22.1		
with other adults	11.9	20.6	15.4		
*Physical activity (h/wk), mean (SD)*	20.4 (15.2)	24.3 (18.2)	24.0 (19.7)	<0.01	0.03
Characteristics of the direct living environment					
*Neighbourhood typology, %*				0.32	0.02
rural	7.3	6.3	4.4		
village-centre	33.0	30.4	25.0		
urban-green	15.0	12.3	10.3		
urban-outside centre	37.8	42.0	47.5		
urban-centre	6.8	9.0	12.5		
*Age of the neighbourhood, %*				0.18	0.23
<1910	8.0	7.7	8.8		
1910-1939	29.4	29.3	22.8		
1940-1969	41.5	35.7	39.0		
1970-1984	18.7	24.9	27.2		
≥1985	2.5	2.4	2.2		
*Availability cycling paths (km/km* ^*2*^ *), mean (SD)*	1.5 (0.8)	1.6 (1.0)	1.9 (1.1)	0.01	<0.01
*Availability walking paths (km/km* ^*2*^ *), mean (SD)*	1.1 (0.8)	1.3 (1.0)	1.6 (1.1)	<0.01	<0.01
*Availability of sports facilities (#/km* ^*2*^ *), mean (SD)*	0.6 (0.3)	0.6 (0.4)	0.7 (0.4)	0.03	<0.01
*Availability of public natural spaces (km* ^*2*^ */km* ^*2*^ *), mean (SD)*	0.1 (0.1)	0.1 (0.1)	0.2 (0.1)	0.55	0.07
*Distance to a sports facility (km), mean (SD)*	1.7 (1.5)	1.4 (1.5)	0.9 (1.2)	<0.01	<0.01
Motivational and situational factors (% that answered often or always)					
Do the following factors influence whether you choose *this transport mode*^*a*^ when travelling these distances?’					
*The weather*	51.0	36.1	21.3	<0.01	<0.01
*I am used to travelling by this transport mode*	66.7	85.6	71.3	<0.01	0.32
*It depends on whether I feel like using this transport mode*	21.4	28.6	27.9	0.01	0.11
*My health/health in general*	15.5	62.8	57.4	<0.01	<0.01
*Whether there is a cycle parking at my destination*	7.1	13.8	8.1	<0.01	0.69
*Season*				0.96	0.08
Winter	28.7	27.8	18.4		
Spring	21.6	20.8	26.5		
Summer	24.8	26.0	24.3		
Autumn	24.8	25.4	30.9		
Perceived route features (% that answered often or always)					
If you travel to the sports facility within a radius of 7.5 km, do you choose *this transport mode*^*a*^ because you…					
*Safety*					
Consider the road traffic situation unsafe when using the other 2 transport modes^b^	5.2	2.4	4.4	0.02	0.70
Feel unsafe when travelling by the other 2 transport modes^b^ because of criminality	3.4	2.4	2.9	0.34	0.79
*Bother by noise, odour and vibrations*					
Are bothered by noise when travelling by the other 2 transport modes^b^	1.1	2.0	2.2	0.28	0.35
Are bothered by odour when travelling by the other 2 transport modes^b^	1.4	1.8	2.2	0.56	0.49
Are bothered by vibrations when travelling by the other 2 transport modes^b^	1.1	1.5	1.5	0.65	0.76
Do the following factors influence your choice of using this transport mode when you travel these distances?					
*Route convenience*					
Whether the distance to my destination is shorter	15.7	38.1	44.1	<0.01	<0.01
Whether it takes less time to reach my destination	54.0	44.8	39.7	<0.01	<0.01
Whether my destination is easy to reach	44.2	52.1	51.5	0.01	0.14
Whether I encounter a lot of traffic lights	8.4	11.8	10.3	0.09	0.50
Whether I encounter obstacles aimed at speed reduction (such as bumps in the road or road narrowings)	6.4	9.0	8.1	0.13	0.49
Whether I am forced to make a detour to reach my destination would I use either of the other 2 transport modes^b^	6.8	15.3	13.2	<0.01	0.02
*Visual aspects*					
What I see/encounter during the trip	4.3	14.0	13.2	<0.01	<0.01
Who I see/encounter during the trip	3.4	12.0	9.6	<0.01	<0.01

yrs = years; SD = standard deviation; h/wk = hour per week; wk = week; km = kilometre; # = number

^a^In this question, the interpretation of ‘this transport mode’ depends on the categorisation of the respondent into a transport mode (i.e. car user, cyclist, walker). If someone was a car user, this transport mode was replaced by ‘to use the car’; if someone was a cyclist, it was replaced by ‘to cycle’; and if someone was a walker, it was replaced by ‘to walk’

^b^If someone was categorised as a car user, the 2 other transport modes are cycling and walking; if someone was a cyclists, the other 2 transport modes are using the car and walking, if someone was a walker the other transport mode are using the car and cycling

If participants indicated that they used multiple transport modes when travelling to a sports facility, they initially answered the 13 questions for each transport mode, because the questions were formulated from the perspective of one transport mode. For example, if someone was categorised as a car user, the following question was asked: ‘If you travel to the sports facility within a radius of 7.5 kilometres, do you choose *to use the car* because you think traffic safety is inadequate when travelling *by bicycle or foot*?’ When someone was categorised as a cyclist or walker, ‘to use the car’ in the question was replaced by ‘to cycle’ or ‘to walk’. The part ‘by bicycle or on foot’ in the previous question was then replaced by ‘by car or on foot’, or by ‘by car or bicycle’, respectively. For the statistical analysis, we only used the answers to the questions that belonged to the transport mode categorisation of a participant. The answers for every item were dichotomised into 0 (‘(almost) never’ and ‘sometimes’) and 1 (‘often’ and ‘always’).

## Covariates

### Individual characteristics

Gender, age, educational level, household composition, and physical activity level were obtained from the questionnaire. Educational level was categorised into: low (primary school and lower general secondary education), medium (intermediate vocational education, higher general secondary education, and pre-university education), and high (higher vocational education and university, reference). Household composition was categorised into: living alone, with a partner, with children under 18, with other adults (parents, children age 18 and older, or other adults; reference). Physical activity was assessed with the validated ‘Short QUestionnaire to ASsess Health-enhancing physical activity’ (SQUASH), which contains questions about multiple activities including commuting, household, leisure time and sport activities referring to a normal week in the past months [16, 17]. Results from the SQUASH were converted to time spent (hours per week) on total physical activity [16–18].

### Motivational and situational factors

Motivational and situational factors that could influence transport choice were also measured with the questionnaire (see Table 1 for the 10 questions). As was the case for the questions regarding route features, we only used the answers to the questions that belonged to the transport mode categorisation of a participant. For example, if someone was categorised as a car user, the following question was asked: ‘Do the following factors influence whether you choose *to use the car* when travelling these distances?’ If someone was categorised as a cyclist or walker, *‘to use the car’* in the question was replaced by *‘to cycle’* or *‘to walk’*. Answers were rated on a four-point category scale (‘(almost) never’, ‘sometimes’, ‘often’, ‘always’). The answers for every item were dichotomised into 0 (‘(almost) never’ and ‘sometimes’) and 1 (‘often’ and ‘always’). Because of possible autocorrelation, we checked how these 10 items were correlated with each other (Additional file 1: Table S1). Because of a lack of power, we set the threshold of the Spearman’s Rho at ≥0.5 instead of ≥0.8 to exclude correlated items. Additional file 1: Table S1 shows that multiple items were correlated. As a consequence, some items were excluded, which is described in Additional file 1. The remaining items were ‘the weather’, ‘I am used to travelling by this transport mode’, ‘I feel like using this transport mode’, ‘my health/health in general’, and ‘cycle parking at destination’. The answers for these items were dichotomised into 0 (‘(almost) never’ and ‘sometimes’) and 1 (‘often’ and ‘always’).

Finally, the season was derived from the date the questionnaire was filled in. Seasons were categorised into: winter, spring, summer, and autumn (reference).

### Characteristics of the living environment

In the Netherlands, postal codes consist of a six-digit postal code starting with four numbers (four-digit postal code), followed by two letters (six-digit-postal code). The six-digit postal code reflects a smaller area within the four-digit postal code, and is thereby more specific than the four-digit postal code. The surface of the four-digit and six-digit areas differs across the Netherlands as will be illustrated next. There are 4000 four-digit postal codes, representing on average 1,772 households each. In urban areas, this four-digit postal code represents only one neighbourhood, whereas in rural areas this postal code can represent a whole village. Each six-digit postal code represents on average 15 to 20 households. In our study, the six-digit postal code of each respondent’s home was available.

To determine neighbourhood typology, we merged our dataset with a dataset from ABF Research (2009) using the four-digit postal code. The data source (ABF Research) provided five different typologies based on density, accessibility/connectivity, land use mix and quality of buildings: 1) Urban – centre, 2) Urban – outside centre, 3) Urban – green, 4) Village-centre, and 5) Rural [19].

For the following characteristics of the living environment, we used the six-digit postal codes of the home address in ArcGIS 10.1.

Another proxy to characterise the neighbourhood was determined: age of the respondents’ neighbourhood. In the Netherlands, the layout of a neighbourhood depends highly on the period in which a neighbourhood was built. Therefore, we categorised the six-digit postal code areas into the following historical periods of urban planning: <1910, 1910–1939, 1940–1969, 1970–1984, ≥1985. We obtained the age of all buildings from the Dutch Registration of Addresses and Buildings (in Dutch: “Basisregistratie Adressen en Gebouwen (BAG)”) [20]. First, we categorised the buildings into the historical periods. Then, the historical period in which most buildings were built was assigned to that area. If the number of buildings between historical periods were equal, the oldest historical period was assigned.

Availability of cycling and walking paths were calculated by summing up the total length of the paths within a circle with a 7.5-km radius originating from the midpoint of the six-digit postal codes of the home addresses (hereafter: living environment). OpenStreetMap [21] was used, which gives detailed information about separate walking and cycle paths. For 10 % of the participants, a proportion of the surface of the living environment was located outside the borders of the Netherlands. As we had no information available about environmental characteristics outside the borders, the length of cycling and walking paths was divided by the surface that was located within the Netherlands.

The availability of sports facilities was assessed by counting the number of sports facilities within the living environment by using the Sports Accommodation Monitor (in Dutch: “Accommodatie Monitor Sport) (Additional file 2: Table S2.1, Table S2.2). Because practicing sports like Nordic walking or mountain biking can take place in public natural spaces, the availability of public natural spaces was assessed by calculating the squared kilometres of public natural spaces within the living environment. The surface of the public natural spaces was obtained from a map of TOP10NL [22], and the function from a map of Statistics Netherlands [23]. The availability of sports facilities and natural public spaces was then corrected for the surface that was located within the Netherlands as described above (calculation density walking/cycling paths).

The distance to the nearest sports facility was assessed by calculating the straight-line distance using the midpoints of the six-digit postal codes of the home address and the sports facility. Sports accommodations and the types of sports that were available per accommodation were registered in the Sports Accommodation Monitor (Additional file 2: Table S2.1, Table S2.2). The sport that the respondent was practicing was obtained from the questionnaire. The facility that offered the sport they were practicing and was closest to the home address was then linked to the information of the respondent in 1187 of 1190 cases. This also included public natural spaces for sports such as running and cycling (see Additional file 2: Table S2.1, Table S2.2 for classification and an explanation of the sports and the accompanying facilities).

### Statistical analysis

Six participants had missing values on individual characteristics and 56 participants had missing values on the distance to a specific sports facility (including the three participants who could not be linked to a sports facility). Finally, 10 participants with extreme values on time spent on physical activity (>112 hrs/wk) were excluded, leaving 1118 participants for the analysis (Fig. 1). Descriptive statistics were carried out for study characteristics. The differences of study characteristics between transport modes were examined with an ANOVA for continuous variables and a χ^2^ test for categorical variables (P < 0.05).

To examine the association between perceived route features and transport choice, we used logistics regression analysis resulting in odds ratios (OR) and their confidence intervals (95 % CI) of cycling versus car use and walking versus car use. The ORs can be interpreted as the likelihood of an average person in our dataset choosing active transport use over car use for trips up to 7.5 kilometres. The ORs were adjusted for individual characteristics, motivational and situational factors and characteristics of the living environment, as previous literature reported that these can influence transport use [9–12]. The 13 perceived route features were entered separately in the model. The statistical analyses were carried out in SAS statistical software, version 9.3.

## Results

### Study characteristics

Table 1 presents characteristics of the participants and the direct living environment and it presents the motivational and situational factors and perceived route features that could influence transport choice when travelling to a sports facility.

Cyclists (mean: 46 yrs) were younger than car users (mean: 48 yrs, p = 0.01). Walkers (p = 0.03) and cyclists (p < 0.01) had a different household composition as compared to car users, i.e. walkers lived alone more often (26.5 % vs. 17.3 %) and cyclists lived more often with other adults (20.6 % vs. 11.9 %), whereas car users more often had children (31.9 %_car_ vs 20.8 %_cyclists_, 22.1 %_walkers_). Both cyclists (24.3 h/wk, p < 0.01) and walkers (24.0 h/wk, p = 0.03) were more physically active than car users (20.4 h/wk).

Walkers lived more often in urban- (outside-) centre areas than car users (47.5% vs. 37.8 %), and had a higher availability of cycling paths (1.9 km/km^2^ vs 1.5 km/km^2^) and walking paths (1.6 km/km^2^ vs 1.1 km/km^2^), sports facilities (0.7 #/km^2^ vs 0.6 #/km^2^) and public natural spaces (0.2 km^2^/km^2^ vs 0.1 km^2^/km^2^) (p ≤ 0.02), and they lived closer to a sports facility (0.9 km vs 1.7 km, p ≤ 0.07). Cyclists also had a higher availability of cycling paths (1.6 km/km^2^ vs 1.5 km/km^2^) and walking paths (1.3 km/km^2^ vs 1.1 km/km^2^), sports facilities (0.6 #/km^2^ vs 0.6 #/km^2^) and public natural spaces (0.1 km^2^/km^2^ vs 0.1 km^2^/km^2^), and they also lived closer to sports facilities than car users did (1.4 km vs 1.7 km) (p ≤ 0.03).

All situational and motivational factors were rated differently between cyclists (13.8-85.6 %) and car users (7.1-66.7 %) (p ≤ 0.01) except for the season (p = 0.96). Walkers rated only the situational and motivational factors such as ‘the weather’ (21.3 %), and ‘my health’ (57.4 %) differently from car users (51.0 % and 15.5 %, respectively, p < 0.01).

Seven out of thirteen perceived route features were rated differently between cyclists and car users, whereas five out of these seven perceived route features were rated differently between walkers and car users. Car users (5.2 %) more often answered that considering the traffic situation unsafe influenced their transport choice than cyclists did (2.4 %, p = 0.02). Most features of perceived route convenience (i.e. distance, time, easy to reach, and detour) were rated differently by car users (15.7 %, 54.0 %, 44.2 %, 6.8 % respectively) than by cyclists (38.1 %, 44.8 %, 52.1%, 15.3 %, respectively, p ≤ 0.01). Walkers only rated distance (44.1 %), time (39.7 %), and detour (13.2 %, p ≤ 0.01) differently from car users. The proportion of car users answering that the two perceived visual aspects of the route influenced their transport choice (3.4 %, 4.3 %) was lower than that of active transport users (9.6-14.0 %, p < 0.01).

### The association between perceived route features and transport choice

In Table 2, the associations between perceived route features and the odds of cycling and walking as compared to using the car are presented.

**Table 2:** Association^*^ between perceived route features and active transport

	OR (95 % CI) _cycling vs car_	OR (95 % CI) _cycling vs car_
If you travel to the sports facility within a radius of 7.5 km, do you choose this transport mode^a^ because you…		
*Safety*		
Consider the road traffic situation unsafe when using the other 2 transport modes^b^	0.36 (0.15-0.86)	1.45 (0.44-4.80)
Feel unsafe when travelling by the other 2 transport modes^b^ because of criminality	0.71 (0.27-1.87)	1.41 (0.30-6.59)
*Bother by noise, odour and vibrations*		
Are bothered by noise when travelling by the other 2 transport modes^b^	0.86 (0.24-3.12)	1.33 (0.16-11.11)
Are bothered by odour when travelling by the other 2 transport modes^b^	0.75 (0.21-2.68)	1.29 (0.16-10.51)
Are bothered by vibrations when travelling by the other 2 transport modes^b^	0.65 (0.17-2.51)	0.39 (0.04-3.92)
Do the following factors influence your choice of using this transport mode^a^ when you travel these distances?		
*Route convenience*		
Whether the distance to my destination is shorter	2.91 (1.97-4.30)	5.21 (2.85-9.52)
Whether it takes less time to reach my destination	0.54 (0.39-0.75)	0.60 (0.36-1.00)
Whether my destination is easy to reach	1.06 (0.77-1.48)	1.39 (0.82-2.36)
Whether I encounter a lot of traffic lights	0.92 (0.54-1.59)	0.56 (0.22-1.42)
Whether I encounter obstacles aimed at speed reduction (such as bumps in the road or road narrowings)	1.02 (0.57-1.85)	0.50 (0.18-1.40)
Whether I am forced to make a detour to reach my destination should I use either of the other 2 transport modes^b^	1.82 (1.06-3.15)	2.15 (0.91-5.08)
*Visual aspects*		
What I see/encounter during the trip	2.40 (1.27-4.53)	3.34 (1.27-8.76)
Who I see/encounter during the trip	2.77 (1.34-5.72)	3.69 (1.29-10.52)

OR = Odds Ratio indicating the odds to choose active transport modes compared to the car; 95%CI = 95% confidence interval; significance was tested at α = 0.05

*Adjusted for: sex, age, education level, household composition, physical activity, neighbourhood typology, age of the neighbourhood, and length (km) cycling lane (for cyclists), length (km) walking paths (for walkers), number of sport facilities, and square km of public natural spaces per km^2^, and the distance to a sports facility, and factors that influence the transport choice (i.e. the weather, I am used to travelling by this ,transport mode, It depends on whether I feel like using this transport mode, My health/health in general, Whether there is a cycle parking at my destination)

^a^In this question, the interpretation of ‘this transport mode’ depends on the categorisation of the respondent into a transport mode (i.e. car user, cyclist, walker). If someone was a car user, this transport mode was replaced by ‘to use the car’; if someone was a cyclist, it was replaced by ‘to cycle’; and if someone was a walker, it was replaced by ‘to walk’

^b^If someone was categorised as a car user, the 2 other transport modes are by ‘bicycle or on foot’; if someone was a cyclists, the other 2 transport modes are using the car and walking, if someone was a walker the other transport mode are using the car and cycling

Perceiving the road traffic situation as being unsafe was associated with lower odds of cycling (OR: 0.36, 95 % CI: 0.15-0.86), but not with the odds of walking (1.45, 95 % CI: 0.44-4.80).

Three aspects of perceived route convenience were associated with transport choice. ‘The trip taking less time’ was associated with lower odds of cycling (OR: 0.54, 95 % CI: 0.39-0.75) and walking (OR: 0.60, 95 % CI: 0.36-1.00). ‘The distance to their destination being shorter’ was associated with higher odds of both cycling (2.91, 95 % CI: 1.97-4.30) and walking (5.21, 95 % CI: 2.85-9.52). ‘Being forced to make a detour when using the other two transport modes than the chosen transport mode’ was associated with higher odds of cycling (1.82, 95 % CI: 1.06-3.15) and near statistically significant with walking (2.36, 95 %: 0.91-5.08).

Perceived visual aspects, i.e. what and who the participants see/encounter during the trip, were associated with higher odds of both cycling (2.40, 95 % CI: 1.27-4.53; 2.77, 95 % CI: 1.34-5.27) and walking (3.34, 95 % CI: 1.27-8.76; 3.69, 95 % CI: 1.29-10.52).

Perceived route features in terms of safety from criminality, bother by noise, odour, and vibrations, and route convenience aspects such as easy to reach their destination, traffic lights and obstacles, were not associated with cycling or walking.

## Discussion

In this study, perceived route features in terms of traffic safety, some aspects of route convenience, and visual aspects were associated with transport choice when travelling to a sports facility. When traffic safety was perceived as unsafe or taking the car took less time, participants were more likely to choose the car than active transport modes. When the distance by using active transport was perceived shorter, or a detour had to be made when using the car, participants were more likely to choose active transport modes over the car. When participants considered what and who they may see/encounter during the trip to be important when making a transport choice, they were more likely to choose active transport modes over the car.

### Strengths and limitations

In our study, we used a questionnaire that was specially designed to examine transport choice for specific trip purposes (i.e. sports). In addition, we were able to study the association between perceived route features and transport choice independent of individual, motivational and situational factors as well as physical characteristics of the built environment, which have been shown to be associated with active transport [9, 11, 24, 25]. Data were collected for all days during a full year, which enabled correction for seasonal influences. Moreover, data were collected from adults across the Netherlands, which provides a good representation of the Dutch adult population living in different environments.

Due to missing values, 27% (N = 401) of the participants who indicated that they made a short trip to a sports facility (N = 1529, Fig. 1) were not included in the analysis. The largest proportion (85%, N = 339) of the 401 participants with missing values had missing values on the sport they practiced. A possible explanation is that they did not practice any sport but, for example, made trips to a sports facility to watch a game or bring their children. Of the remaining 62 persons with missing values, six persons had missing data on individual characteristics and 56 persons had missing values on the distance to the sports facility. This latter might be due to a lack of information on the exact destination of our respondents. We assumed that they would go to the nearest sports facility linked to the sport they were participating in by means of the Sports Accommodation Monitor. It might be that their destination, i.e. sports facility, was not in the Sports Accommodation Monitor or that the sports facility was just outside the range of a 7.5-km radius. Moreover, we used straight-line distances instead of route distances, because of a lack of information of the route taken to their destination. In future, studies should consider gathering information about route distances and destinations (for example by using GPS tracking).

### Putting results into context of the literature

To our knowledge, only a few studies have examined the association between features of the route and active transport use [26–28]. When comparing these studies with our study, it should be kept in mind that the study design in terms of definitions of active transport use, measures of (perceived) route features, setting (city, or a defined radius from the home address), and country (e.g., the Netherlands is densely populated and known for its unique cycling environment [29]), which can be a part of the explanation of differences in findings.

### Traffic safety

When we compare the studies with regard to traffic safety, in the study by Panter and colleagues, less traffic was associated with lower odds of walking to work, which may be explained by the fact that walking to work is probably more prevalent in built-up areas where traffic levels are higher [26]. In the study by Titze and colleagues, students who cycled regularly to the university rated the traffic safety lower as compared to non-cyclists. This may seem odd, but it might be explained by the non-awareness of dangers by non-cyclists who may not have cycled the route to the university, whereas regular cyclists probably are aware of the dangers [27]. In our study, traffic safety was inversely associated with cycling as compared to using the car indicating that those who travel by car to a sports facility more often perceive the traffic situation as unsafe as compared to those who travel by bicycle. This suggests that the experience of safety may be different between trip purposes, as in our study where people travel for recreational purposes (i.e. sports), thinking the traffic situation being unsafe was associated with passive transport use, whereas in the other two studies high traffic volume or unsafe traffic situations did not stop the participants from walking or cycling to work or the university.

### Route convenience

With regard to perceived route convenience, Panter and colleagues found higher odds for both ‘1-149 min/wk’ cycling and ‘ ≥150 min/wk’ cycling if participants indicated that ‘there are convenient routes for cycling’ [26]. In the Norfolk study, both men and women who lived a relatively short distance from work were more likely to actively commute, whereas having a main or secondary road on the route to work was associated with a decreased likelihood of active commuting [28]. The latter may be explained by safety concerns as the presence of these roads could reflect unpleasant traffic interaction (busy, noise, high speeds) when actively commuting [28], or by the fact that access to their destination is simply easier by car because of these roads. In our study, we also found aspects of route convenience (i.e. distance, time and detour) to be associated with active transport use. However, other aspects of perceived route convenience such as easy to reach, traffic lights, and obstacles to reduce speeding were not associated with active transport use. Similarly, Titze and colleagues found no association between perceived traffic flow (continuous cycling, presence of traffic lights) and connectivity (shortcuts, quickness compared to driving a car) and active commuting [27]. From a previous review with objectively measured neighbourhood characteristics, it was shown that residents from communities with higher density, greater connectivity and more land use mix more often used active transport than low-density, poorly connected, and single land use neighbourhoods [25], raising the expectations that perceived route convenience, such as connectivity and easy to reach could be of importance in using active transport. This mismatch between perceived and objective accessibility measures has previously been shown [30, 31]. In addition, a recent study within the AVENUE project has shown that perceived accessibility, irrespective of objective accessibility, was strongly associated with transport choice for trips with the purpose of shopping, sports or public natural spaces (Scheepers CE, Wendel-Vos GCW, van Kempen EEMM, de Hollander EL, van Wijnen HJ, Maas J, den Hertog FRJ, Staatsen BAM, Stipdonk HL , Int Panis LLR, van Wesemael PJV, Schuit AJ: Perceived Accessibility is An Important Factor in Transport Choice — Results from the AVENUE Project, Submitted). This indicates that perceived environmental characteristics cannot be translated to objectively measured environmental characteristics. This can be illustrated by case studies [32, 33]. One study found that cycling commuters were more likely to take a route that was bumpy, but quiet and green, instead of taking the separate cycling lane designed by urban planners [33]. Another case study including different neighbourhoods in Amsterdam, the Netherlands, showed that individuals perceived the distance to be shorter if many people and less traffic were observed along the route [32]. Taking all of these aspects into account, this indicates that determining the contribution of perceived features and the contribution of objective features in stimulating active transport use is difficult as subjective and objective features interact with each other. Future research should incorporate these aspects in order to guide policy makers and urban planners in the development of measures to stimulate active transport use.

### Visual aspects

With regard to perceived visual aspects, attractiveness was positively associated with irregular cycling but not with regular cycling compared to non-cycling in the study by Titze et al. [27]. In a previous study, it was shown that physical activity in general is positively associated with attractiveness [34]. Since cycling to work is different from cycling during leisure time (recreation), it seems reasonable that attractiveness is more important for irregular cyclists than for regular cyclists [27]. In our study, visual aspects of the route were positively associated with both cycling and walking as compared to using the car when travelling to a sports facility. This might be explained by visual aspects being more important when travelling for recreational purposes (i.e. to a sports facility) than for school purposes. To illustrate further the importance of the trip purpose in the association between perceived environmental factors and active transport use, previous studies have shown that different perceived environmental factors were associated with different types of walking [24], and the strength of the association of perceived accessibility differed per trip purpose (i.e. shopping, work, public natural spaces and sports facility) (Scheepers CE, Wendel-Vos GCW, van Kempen EEMM, de Hollander EL, van Wijnen HJ, Maas J, den Hertog FRJ, Staatsen BAM, Stipdonk HL, Int Panis LLR, van Wesemael PJV, Schuit AJ: Perceived Accessibility is An Important Factor in Transport Choice — Results from the AVENUE Project, Submitted). Moreover, from exploratory analysis in the AVENUE study, we found that persons who indicated that they travelled for sports and working purposes, the transport choice differed in 33% of the cases. For sports and shopping purposes, the difference was 41% and for sports and public natural spaces purposes 48%. Thus, it is very well possible that not only the experience and importance of route features differ per trip purpose, but that people also choose different transport modes to travel to different destinations. Therefore, future research should take trip purpose into account when examining the associations between route features and active transport use. Consequently, when developing policy measures to stimulate active transport, the target group (workers, students or sports practitioners) should be taken into account.

## Conclusions

For Dutch adult sports practitioners, perceived route features in terms of traffic safety, convenience, and visual aspects were associated with transport choice when travelling to a sports facility. This suggests that the perception of different route aspects should be considered when developing measures for stimulating active transport use among sports practitioners.

### Data Availability

The authors confirm that, for approved reasons, some access restrictions apply to the data underlying the findings. The data used in this study are available upon request. There are some legal restrictions preventing the publication of data as Supporting Information files or in a public repository, as data are from an existing online panel. This online panel is the property of a third party (Ipsos (http://www.ipsos.com/)). To request the data, the following persons can be contacted: info@rivm.nl (Centre for Nutrition, Prevention and Health Services), Wanda Wendel-Vos (wanda.vos@rivm.nl), Eline Scheepers (eline.scheepers@rivm.nl).

## Additional files

Additional file 1: Table S1. 1Correlations between factors that could influence the choice of transport. Additional file 2: Table S2. 1Calculating the distance to a sports facility according to different types of sport.

## Abbreviations

AVENUE: impActs of actiVE traNsport in Urban Environments
BAG: Dutch Registration of Addresses and Buildings (in Dutch: “Basisregistratie Adressen en Gebouwen)
CI: Confidence interval
OR: Odds ratio
PA: Physical activity
SQUASH: Short QUestionnaire to ASsess Health-enhancing physical activity
